# EUSKOR: End-to-end coreference resolution system for Basque

**DOI:** 10.1371/journal.pone.0221801

**Published:** 2019-09-12

**Authors:** Ander Soraluze, Olatz Arregi, Xabier Arregi, Arantza Díaz de Ilarraza

**Affiliations:** 1 Computer Languages and Systems Department, University of the Basque Country, Donostia-San Sebastian, Spain; 2 Computer Architecture and Technology Department, University of the Basque Country, Donostia-San Sebastian, Spain; STL UMR8163 CNRS, FRANCE

## Abstract

This paper describes the process of adapting the Stanford Coreference resolution module to the Basque language, taking into account the characteristics of the language. The module has been integrated in a linguistic analysis pipeline obtaining an end-to-end coreference resolution system for the Basque language. The adaptation process explained can benefit and facilitate other languages with similar characteristics in the implementation of their coreference resolution systems. During the experimentation phase, we have demonstrated that language-specific features have a noteworthy effect on coreference resolution, obtaining a gain in CoNLL score of 7.07 with respect to the baseline system. We have also analysed the effect that preprocessing has in coreference resolution, comparing the results obtained with automatic mentions versus gold mentions. When gold mentions are provided, the results increase 11.5 points in CoNLL score in comparison with results obtained when automatic mentions are used. The contribution of each sieve is analysed concluding that morphology is essential for agglutinative languages to obtain good performance in coreference resolution. Finally, an error analysis of the coreference resolution system is presented which have revealed our system’s weak points and help to determine the improvements of the system. As a result of the error analysis, we have enriched the Basque coreference resolution adding new two sieves, obtaining an improvement of 0.24 points in CoNLL *F*_1_ when automatic mentions are used and of 0.39 points when the gold mentions are provided.

## Introduction

Coreference resolution consists of identifying textual expressions (mentions) that refer to real-world objects (entities) and determining which of these mentions refer to the same entity. Coreference resolution is helpful in NLP applications where better comprehension of the discourse leads to improved performance. Information Extraction, Question Answering, Machine Translation, Sentiment Analysis, Machine Reading, Text Summarization and Text Simplification, among others, can benefit from coreference resolution.

It is very common to divide the task of coreference resolution into two main subtasks: mention detection and resolution of references [1]. Mention detection is concerned with identifying potential mentions of entities in the text and resolution of references involves determining which mentions refer to the same entity.

In less-resourced languages it is particularly challenging to develop highly accurate tools for tasks like mention detection and coreference resolution. Besides, it is complex to create completely language-independent systems, whereas taking into account the characteristics of a language benefits performance of these tasks. In this scenario, a possible solution is to use a state-of-the-art system with a flexible modular architecture and adapt it to resolve coreference in the new language to be treated. In our particular case, we have adapted the Stanford Coreference resolution system [2] to Basque. The process we carried out demonstrates that using a modular architecture facilitates the development of robust coreference resolution systems for any language with different characteristics to the language for which the system was originally created.

This paper is structured as follows. After reviewing related work, we describe the most important characteristics of Basque and the challenges they present for coreference resolution. Then, the architecture of EUSKOR, an end-to-end coreference resolution, is presented and the adaptation process explained. After that, the experiments carried out to evaluate the coreference resolution system are described. Then an error analysis of the system is presented, and proposed improvements explained. In section “Comparison of coreference system for Basque” a comparison of our rule-based system with two other systems for Basque is presented. Finally, conclusions and future work are discussed.

## Related work

Much attention has been paid to the problem of coreference resolution and many evaluation campaigns focusing on the topic have been undertaken in the last decades, from MUC-6 [3] in 1995 to the CoNLL shared task in 2012 [4]. Although coreference resolution in English texts was the main focus of the sixth and seventh Message Understanding Conferences [3, 5], studies of coreference resolution in languages other than English were published in this period, including Japanese [6], Spanish [7], French [8] or Portuguese [9]. A new trend towards multilinguality in the field was established by [10] or [11].

The Automatic Content Extraction (ACE) program [12] aimed to identify certain types of coreference relations between a predefined set of entities for Arabic and Chinese in addition to English.

SemEval-2010 Task 1 [13] focused on coreference in multiple languages (English, Dutch, German, Italian, Spanish and Catalan) and it addressed interesting questions, such as: i) to what extent is it possible to implement a general coreference resolution system that is portable to different languages? ii) how much language-specific tuning is necessary to achieve this goal? and iii) how can morphology, syntax and semantics help to solve coreference in each language?

One year later, in the CoNLL 2011 shared task [1], participants had to model unrestricted coreference in the English-language OntoNotes corpora [14]. The system that obtained the best results in the 2011 edition was the Stanford system presented in [2]. The CoNLL 2012 shared task [4] focused on coreference resolution in an English, Chinese and Arabic multi-lingual setting. The Stanford system was used by six participants [15–20]. Depending on the language to be treated, participants used different strategies, e.g., [15] seek to improve the multi-pass sieve approach by incorporating lexical information using machine learning techniques. They employed different sieves depending on the language. [16] took into consideration the special characteristics of each language and used the Stanford system to generate mention link candidates, which were then reranked by a supervised model.

Recently, different approaches have been presented for coreference resolution in English. Latent antecedents [21, 22] or neural models [23, 24] have gained popularity and achieved state-of-the art results. Hybridisation of techniques has been also proposed, for example in [25], which presents an hybrid architecture of their Stanford system. This approach incorporates both rule-based and statistical machine learning sieves.

Interest in coreference resolution in languages other than English has been increasing in the last few years [26, 27], and because of this many papers about adaptations of coreference resolution systems to a language other than the one for which they were created have been published. The Beautiful Anaphora Resolution Toolkit (BART) [28] has been adapted to many languages: originally created for English, its flexible modular architecture facilitates its portability to other languages. There has therefore been a lot of work on extending the BART coreference toolkit to languages other than English including Italian [29], German [30], Polish [31], Arabic and Chinese [32], and Indian languages [33].

## Basque characteristics for coreference resolution

Basque is a language isolate. Its grammar differs considerably from that of the languages spoken in its neighbouring regions. It is an agglutinative, head-final, pro-drop, free-word-order language. There is no grammatical gender in the nominal system. There are no distinct forms for third person pronouns, instead of which demonstratives are used [34]. All these characteristics make coreference resolution for Basque more challenging in some respects.

On account of Basque’s agglutinative nature, a given lemma of nouns and adjectives can take many different word forms, depending on case (genitive, locative, etc.) and number (singular, plural, indefinite). Therefore looking only for the given word form is not enough to resolve coreference in Basque when string matching techniques are applied.

Unlike English, Basque is head-final, i.e. the head of a phrase follows its complements, whereas English is head-initial, with the head of a phrase preceding its complements. Because the correct identification of the head of mentions is of fundamental importance in coreference resolution, the language’s head directionality must be considered.

As for word order typology, Basque is known to be a free word order language. Consequently, the same sentence can be written in different manners. For example, the sentence *Jonek liburua irakurri du* “John has read the book” is in neutral word order (SOV) but it has five more variations (SVO, OVS, OSV, VOS, VSO) owing to free word order.

The free-word-order nature of Basque can make the correct identification of syntactic function more complex as it becomes more ambiguous. This has a direct effect on coreference resolution, since the syntactic function is a feature that is commonly used in the resolution phase.

Basque is a pro-drop language in which zero subject pronouns can be inferred from the verb form. Basque also allows direct and indirect objects to be omitted. Basque is therefore said to be a three-way pro-drop language. As a consequence of this characteristic, many pronouns that need to be resolved are omitted.

Finally, the lack of grammatical gender in the nominal system makes it impossible to use gender as a feature in the coreference resolution process, which has been proven particularly useful in the resolution of pronouns in some languages. The animacy feature cannot be used for pronoun resolution either, because Basque pronouns do not have such feature.

## Mention structures in Basque

EPEC (Reference Corpus for the Processing of Basque) [35] is a 300,000 word sample collection of standard written Basque that has been manually annotated at different levels (morphology, surface syntax, phrases, etc.). The corpus is composed of news published in *Euskaldunon Egunkaria*, a Basque language newspaper. It is aimed to be a reference corpus for the development and improvement of several NLP tools for Basque. Mentions and coreference chains have been annotated semi-automatically based on previous layers in a 46,383-word subset of the EPEC corpus. Automatically annotated mentions obtained by our mention detector [36] were first corrected by linguists; then, coreferent mentions were linked in clusters. It is freely available and can be downloaded at: ixa2.si.ehu.eus/epec-koref/epec-koref_v1.0.tgz.

With reference to the subtask of mention detection, in this section we establish what mentions we regard as potential ones to be included in a coreference chain based on a linguistically motivated mention classification presented in [37].

1. **Proper nouns**: Structures that have a proper noun as head.
  (a) *[Clinton] itxaropentsu agertu_zen kazetarien aurrean*.
    “[Clinton] appeared hopeful in front of the reporters.”
2. **Pronouns**: In Basque, no separate forms exist for third person pronouns versus demonstrative determiners; demonstrative determiners are used as third person pronouns [34]. Therefore, we mark the mentions formed by demonstratives used as pronouns.
  (b) *LDPko buruek Mori hautatu zuten apirilean Keizo Obuchi orduko lehen ministroa ordezkatzeko, [hark] tronbosia izan ostean*.
    “The heads of LDP chose Mori in April, to replace the Prime Minister Keizo Obuchi, [who (he)] suffered a thrombosis.”
3. **Possessives**: We consider two types of possessives: NPs containing a possessive determiner, even if it is not the head of that NP as in (c), and possessive pronouns as in (d).
  (c) *Epitieren kasuan [[bere] helburua] lortu dezakela dirudi eta baliteke denboraldia Lehen Mailan hastea*.
    “In the case of Epitie, it seems that he could achieve [[his] aim] and possibly start the football season in the Premier League.”
  (d) *Escuderok euskal musika tradizionala eraberritu eta indartu zuen. [Harenak] dira, esate baterako, Illeta, Pinceladas Vascas eta Eusko Salmoa obrak*.
    “Escudero renewed and gave prominence to traditional Basque music. The works Illeta, Pinceladas Vascas and Eusko Salmoa, for example, are [his].”
4. **Verbal nouns**: As in many other languages, verbs can be nominalised in Basque. When the nominalised verb works as the head of the mention (ixtea “closing” in (e)) and takes the corresponding case marking suffix, the whole clause governed by the verbal noun has to be annotated.
  (e) *[Instalazio militarrak ixtea] eskatuko dute*.
    “They will ask for [closing the military installations].”
5. **NPs as part of complex postpositions**: Basque has a postpositional system, and therefore we mark the independent NP preceding a complex postposition. In (f) the postposition is *aurka* (“against”), and we annotate the noun (in his case a proper noun) that precedes it.
  (f) *Joan den astean [Moriren] aurka aurkeztutako zentsura mozioak piztu zuen LDPko krisia*.
    “Last week the vote of no confidence against [Mori] caused the crisis in the LDP.”
6. **NPs containing subordinate clauses**: The head of these mentions is always a noun complemented by a subordinate clause. In (g) the head noun is complemented by a subordinate clause of the type that is, for Basque, called a *complementary clause*. We take the whole stretch of the NP (both the subordinate clause and the head noun) as a mention.
  Relative clauses can also add information to nouns as in (h), in which case the boundaries of the mention are set at the beginning of the relative clause and the end of the NP.
  (g) *[DINAk Argentinan egindako krimenak ikertzeko baimena] eman du Txileko Gorte Gorenak*.
    “The Supreme Court of Chile has given [permission to investigate the crimes DINA committed in Argentina].”
  (h) *[Igandeko partiduak duen garrantzia] dela eta, lasai egotea beharrezkoa dutela esan zuen Lotinak*.
    “Lotina said that it is necessary to stay calm given [the importance that Sunday’s match has].”
7. **Ellipsis**: Ellipsis is a widespread phenomenon in Basque.
  At a morphosyntactical level, a noun ellipsis occurs when the suffixes attached to the word belong to a noun although that noun is not explicit in the word. We recognise this type of ellipsis in the case of verbs that take suffixes indicating noun ellipsis, as in example (i). The POS given by the analyser indicates the presence of an ellipsis, which is implied by the presence of both the verb (*sailkatu zen-* “finished”) and the ellipsis (*-Ø-ak* ‘who…’). In this case Ø refers to someone. All the information corresponding to both units is stored and treated as a noun.
  (i) *[Bigarren sailkatu zenak] segundo bakarra kendu zion*.
    “[Ø who finished in second place] only had a second’s advantage.”
  At sentence level, the subject, object or indirect element of the sentence can be elided. The morphological information about these elements (number, person…) is given by the verb. We do not mark these elliptical pronouns as mentions (j).
  (j) *Ø Ez zuen podiumean izateko itxaropen handirik*.
    “[He] did not have much hope of being on the podium.”
8. **Coordination**: In the case of coordination, nominal groups of a conjoined NP are extracted. We regard as mentions both the nested NPs (*siesta*, “a nap” and *atsedena* “a rest”) and the whole coordinated structure (*siesta eta atsedena* “a nap and rest”).
  (k) *Bazkal ondoren [[siesta] eta [atsedena]] besterik ez zuten egin*.
    “After lunch they did nothing but have a [[a nap] and [a rest]].”
9. **Location Adverbs (LocAdv):** In general, adverbs are not referential, yet location adverbs do have a referential function. Therefore, location adverbs are considered mentions.
  (k) *Futbol jokalariak Biarritzera joatekoak ziren, [han] festa antolatuta baitzuten*.
    “The football players intended to go to Biarritz, because they had a party organised [there].”
10. **Common Noun Phrases (CNP):** Phrases that have a noun as head word.
  (l) *[Langileak] haserre daude hartutako erabakiarekin*.
    “[The workers] are angry with the decision taken.”

## System architecture

**Fig 1:**
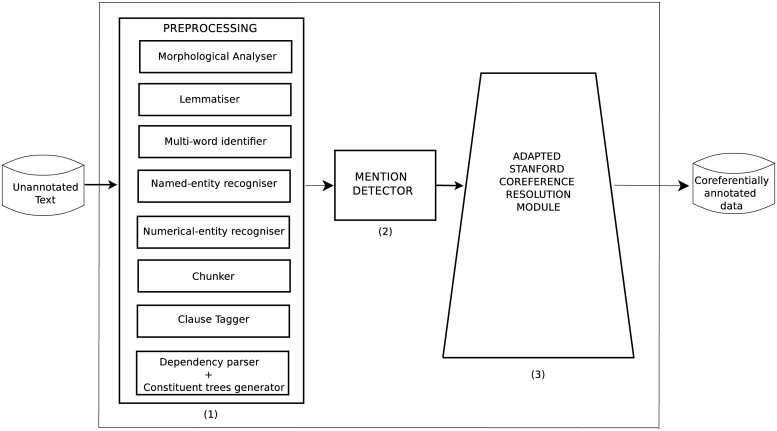
End-to-end coreference resolution for Basque.

In this section, EUSKOR, an end-to-end coreference resolution system is presented. As shown in Fig 1, the system has three main components: i) preprocessing module, ii) mention detector and iii) coreference resolution module.

### Preprocessing

The preprocessing stage prepares the input that the coreference resolution system receives. In this stage, Basque linguistic processors are applied to the text, thus obtaining linguistically annotated data.

The Basque linguistic processors used to create annotations are the following:

1. A morphological analyser that carries out word segmentation and PoS tagging [38].
2. A lemmatiser that resolves the ambiguity caused at the previous phase [39].
3. A multi-word item identifier that determines which groups of two or more words are to be considered multi-word expressions [40].
4. A named-entity recogniser that identifies and classifies named entities (person, organization, location) in the text [41].
5. A numerical-entity recognizer that identifies and classifies numerical entities (date, time, percent, number…) in the text [42].
6. A Basque dependency parser [43]; its output is then used to create constituent trees [44].

### Mention detection

We created a mention detection system [36], defining a set of hand-crafted rules that have been compiled into Finite State Transducers (FST). These FSTs are able to detect complex structures that should be identified as mentions. We defined 12 FSTs, composed of 34 hand-crafted rules using Foma [45].

The system has been developed and evaluated using the subset of the EPEC corpus, annotated for coreference purposes, consisting of 46,383 words with 12,792 mentions. The mention detector has a precision of 74.79, a recall of 72.94 and an F-measure of 73.86 when automatic preprocessing is used and a precision of 84.95, a recall of 86.84 and an F-measure of 85.89 with gold standard data.

### Stanford Coreference resolution module

The Stanford Coreference Resolution module is a deterministic rule-based system which is based on ten independent coreference models or sieves that are precision-oriented, i.e., they are applied sequentially from highest to lowest precision. Each model selects a single best antecedent from a list of previous mentions or declines to propose a solution. Candidates in the same sentential clauses are sorted using left-to-right breadth-first traversal of syntactic trees to favour subjects [46]. Nominal mentions in previous sentences are sorted using right-to-left to favour proximity. In the case of pronominal mentions, candidates in previous sentences are also treated left-to-right traversal in order to favour subjects that are more probable antecedents for pronouns. The sorting of candidates is important, as low quality negatively impacts the coreference links created.

The architecture is highly modular, which means that additional coreference models can easily be integrated. The system implements an entity-centric approach, allowing each coreference resolution decision to be globally informed by previously clustered mentions and their shared attributes. Finally, the lack of language-specific lexical features makes the system easy to port to other languages [2].

**Fig 2:**
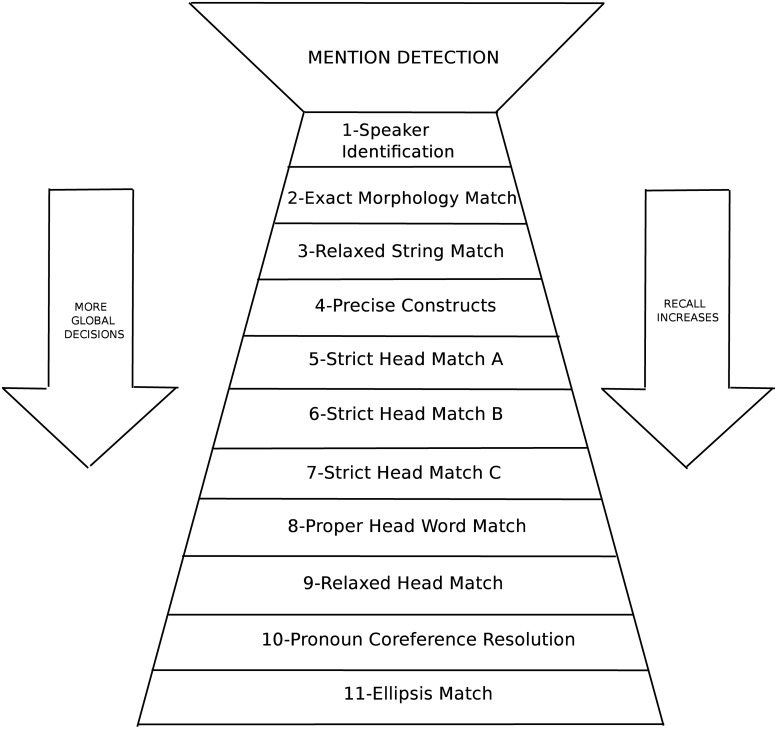
The architecture of the Basque coreference resolution system.

The system has been adapted to resolve coreference in Basque, modifying some sieves and adding new ones. The version presented here is an extension of the system presented in [47]. The architecture of the Basque coreference resolution system is shown in Fig 2, where the order of application of the sieves is indicated. We shall begin by explaining how the original sieves work, discussing the problems found and the adaptations proposed.

(S1) **Speaker Identification:** The sieve identifies speakers and links them with corresponding compatible pronouns. In non-conversational texts, the speakers are identified by searching the subjects of reporting verbs. In conversational texts, speaker information is provided in the dataset.
  **Adaptation**: We have translated the list of reporting verbs and the list of pronouns.
(S2) **Exact String Match:** The Exact String Match sieve links two mentions with exactly the same extent text.
  In (1), the sieve matches the mentions [Milosevicek] and [Milosevicek] “[Milosevic]” and it considers them coreferent, however, [Milosevici] “on [Milosevic]” is not linked.
  (1) [Milosevicek] bere herriaren borondatea errespetatu beharko luke. Joe Lockhardek bozeramailearen ustez, [Milosevicek] lehenbailehen utzi beharko luke gobernua. Etxe Zuriko maizterrak esandakoak presio handiagoa egiten dio [Milosevici] boterea ahal den azkarren utz dezan.
    “[Milosevic] should respect the will of its people. In the opinion of spokesman Joe Lockhard [Milosevic] should leave the government immediately. The words of the occupant of the White House put more pressure on [Milosevic] to leave power as soon as possible.”
  **Problem**: The constraint applied in the Exact String Match sieve is too restrictive in agglutinative languages, as the role of prepositions is played by suffixes added to word forms. Consequently, two mentions that refer to the same entity but differ in their word forms are not considered coreferent. That is the case of the mention [Milosevici] in example (1), it is not considered coreferent with [Milosevicek] as their extent text differs.
  **Adaptation**: We created a specialisation of the Exact String Match sieve, named the **Exact Morphology Match** sieve, and substituted it in the adapted version of the coreference resolution system. This sieve takes into account morphological features of mentions to consider if they corefer or not. Two mentions are linked if i) the lemmas of each word in both mentions are identical, and ii) their number and definiteness are equal (or unknown in one of the mentions). Number attributes can take the values of singular, plural or unknown and definiteness can be finite, indefinite or unknown (treated as wildcards, i.e., they can match any other value).
  It is important that two mentions fulfil these three constraints at the same time because even if one or two are fulfilled, the mentions may not be coreferent as the examples in Table 1 illustrate.
  The first and second mentions, *txori politak* and *txori politekin*, are coreferent because the conditions are fulfilled: same lemmas, same number and same definiteness. Although the first and third mentions are identical strings, they are not coreferent. The first mention *Txori politak* represents a plural mention in the absolutive case, whereas the same string in the third row represents a mention in the ergative singular (this morphological information has been extracted previously with help from the context). The first and fourth mentions have the same lemma and number but their definiteness differs (the first is definite while the second is indefinite), so they cannot be considered coreferent.
  After substituting the Exact Morphology Match sieve for the Exact String Match sieve, the three mentions in example (1) are considered coreferent.
(S3) **Relaxed String Match:** This sieve considers two mentions as coreferent if the strings obtained by dropping the text following their head words, such as, relative clauses and participial postmodifiers (clauses headed by participial form of the verb) are identical.
  **Problem:** In English, relative clauses follow the head word, however, in Basque they can follow or precede the head word. In the following example, the two possibilities in Basque for the relative clause “[Bill Clinton who accepted the new law] appeared hopeful in front of the reporters.” are presented. Although, the following two examples in (2) are correct, the (a) case is the most common in Basque.
  (2) a [Lege berria onartu duen Bill Clinton] itxaropentsu agertu zen kazetarien aurrean.
    b [Bill Clinton zeinak lege berria onartu duen] itxaropentsu agertu zen kazetarien aurrean.
  Similarly to relative clauses, participials in Basque can appear in two manners, however, they mostly precede the noun and, ocassionally, phrases can appear apposited to the right of the noun phrase.
  **Adaptation:** The Relaxed String Match sieve has been modified to take relative clauses and participials that precede the noun into account. The adapted sieve is also able to drop the text of relative clauses and participials preceding the head word. To consider two mentions coreferent the compared mentions also have to fulfil the same morphological constraints applied in The Exact Morphology Match sieve.
  After the adaptation, the sieve is able to link the two mentions in example (3).
  (3) [Gailurretako Rallyan] iragan urteko balentria handia errepikatu nahi izango dute pilotu zuberotarrek. [Igandean bukatuko den Gailurretako Rallyan] lehiakide zorrotzenekin topo egingo dute.
    “In [Rally des Cimes] Soule pilots will want to repeat last year’s great bravery. [In Rally des Cimes which ends on Sunday] they will meet the ablest opponents.”
(S4) **Precise Constructs:** The Precise Construct sieve links two mentions, if any of the conditions below is fulfilled:
  - *Appositive*: The two mentions are in an appositive relation.
  - *Predicative nominative*: The two mentions are in a copulative construction.
  - *Acronym*: One of the mentions is an acronym of the other mention and both are tagged as a proper noun. The algorithm to detect acronyms marks one mention as an acronym of the other if its text equals the sequence of upper case characters in the other mention.
  - *Demonym*: One of the mentions is a demonym of the other. For demonym detection a static list of countries and their corresponding demonyms extracted from Wikipedia is used.
  - *Role appositive*: The candidate antecedent’s head word is a noun and it appears as a modifier in a Noun Phrase whose head is this mention. For example, [[the singer] Michael Jackson].
  - *Relative pronoun*: The head of the antecedent Noun Phrase is modified by a relative pronoun mention, e.g., [the business street [which] has already formed in the Waitan district].
  **Problem:** The rules used to detect appositive and predicative nominative structures are not the most suitable for Basque. The algorithm to detect acronyms does not consider that acronyms can appear declined. Furthermore, the way in which our gold-standard corpus of mentions and coreference chains was annotated does not treat role appositives and relative pronoun structures as mentions. For example, in the case of Michael Jackson abeslaria “the singer Michael Jackson”, the whole structure is considered one mention [Michael Jackson abeslaria] and not two mentions [[the singer] Michael Jackson] as in English, i.e., [abeslaria] is not treated as a mention.
  **Adaptation:** A detector of appositive and predicative nominatives for Basque [48] has been integrated in the preprocessing pipeline. The information obtained by the detector is then provided to the coreference resolution system and links mentions that are considered appositive or predicative nominatives. In addition, a Basque lexicon with 373 entries listing names of nationalities and demonyms provided by the Basque language academy “Euskaltzaindia” has been included in the coreference resolution system.
  The algorithm to detect acronyms has been changed to recognise declined acronyms by considering a mention an acronym of another if its lemma equals the sequence of upper case characters in the other mention. Example 4 illustrates the need to compare acronym lemmas rather than its text. If acronym text [AABek] “[CA]” were compared with [Amnistiaren Aldeko Batzordeak] “[Commission for Amnesty]”, the two mentions would not be considered coreferent.
  (4) Europako Giza Eskubideen Agiria ez dela betetzen salatuko dute [AABek] Nizan. Europako Batasuneko estatuburuen bilerara joango dira [Amnistiaren Aldeko Batzordeak] bertan onartuko den Giza Eskubideen Europako Agiria Euskal Herrian praktikara ez dela eramaten salatzera.
    “[CA] will denounce in Nice that the European Convention on Human Rights is not met. [Commission for Amnesty] will go to the meeting of state heads of the European Union to denounce that the European Convention on Human Rights to be accepted there is not put into practice in the Basque Country.”
  The original English static list of countries and their corresponding demonyms has been replaced by a Basque version to identify demonyms. The list has been created using the 38th rule of “Euskaltzaindia” (Royal Academy of the Basque Language).
  Finally, the Role Appositive and Relative Pronoun constraints have been deactivated. Role appositives are treated in sieve 9 (S9).
(S5-S7) **Strict Head Match A-C:** The Strict Head Match sieve links two mentions if all the following constraints are satisfied at the same time:
  - *Entity head match*: The mention head word matches any head word in the antecedent entity.
  - *Word inclusion*: All the non-stop words in the current entity to be solved are included in the set of non-stop words in the antecedent entity.
  - *Compatible modifiers only*: The modifiers of the mention to be resolved are included in the modifiers of the antecedent candidate. As modifiers, nouns and adjectives are considered.
  - *Not-i-within-i*: The two mentions are not in i-within-i construct, i.e., one cannot be a child NP in the other’s NP constituent.
  The variants B and C of the Strict Head Match are relaxations of the constraints explained above. Strict Head Match B (S6) removes the constraint *compatible modifiers* and Strict Head Match C (S7) removes *word inclusion*.
  **Problem:** This sieve is not suitable for direct application in agglutinative languages because, in three of the four constraints word forms are compared.
  **Adaptation:** We have modified the *Entity head match*, *Word inclusion* and *Compatible modifiers only* constraints. The adapted constraints compare lemmas instead of word forms. In addition, in *Entity Head Match* the compared head words must fulfil number and definiteness agreement.
  After the adaptation of the three constraints, the sieve correctly links the mentions in example (5) as all the constraints are fulfilled. The mentions in example (6) are not linked. They are not coreferent, since even if the *Entity Head Match* constraint is fulfilled, the *Word inclusion* and *Compatible modifiers only* constraints are not satisfied.
  (5) [Euskalteleko kirol zuzendari Julian Gorospek] emaitza onak lortzeko moduan daudela uste du. [Julian Gorosperentzat] Tourrean izateko aukera gutxi zuten, eta beraz, ondo hartu du berria.
    “[Euskaltel sporting director Julian Gorospe] thinks they can get good results. For [Julian Gorospe] they had little chance of being on the Tour, and therefore, he took the news well.”
  (6) Eurokopako atezain onena bilakatu da Toldo, eta Italian heroi nazionala da. Merezita lortu du [Fiorentinako atezainak] gailurrera iristea. Peruzziri ere zor dio Toldok zerua ukitu izana. Buffonek titulartasuna kendu izana ez du ondo irentsi [Inter Milaneko atezainak] eta uko egin zion Eurokopari.
    “Toldo has become the best goalkeeper in the European Championship, and he is a national hero in Italy. [The Fiorentina goalkeeper] deserved to reach the top. Toldo is also indebted to Peruzzi for having touched the sky. [The Inter Milan goalkeeper] did not Buffon’s robbing him of the championship title well and rejected the European Championship.”
(S8) **Proper Head Word Match:** This sieve considers two mentions coreferent if the following constrains are satisfied:
  - Both mentions are headed by proper nouns and the head word is the last word of the mention.
  - Not-i-within-i
  - *No location mismatches*: The modifiers of two mentions do not have different location named entities, other proper nouns, or spatial modifiers.
  - *No numeric mismatches*: The mention to be resolved does not have a number that does not appear in the antecedent mention.
  **Problem:** Basque is a free word-order language, consequently, the head word of a mention should not necessarily appear in the last position.
  **Adaptation:** We changed the first constraint to permit the head word of the mention to appear in other positions apart from the last position. In addition, lemmas instead of word forms are used to compare head words and modifiers.
  In example (7), the adapted sieve considers the mentions [Batistutarentzat] “for [Batistuta]” and [Gabriel Batistuta argentinarrak] “[Argentinian Gabriel Batistuta]” (where the head word is Batistuta) coreferent, but discards mentions in example (8) as they have a different spatial modifier.
  (7) [Batistutarentzat] ez da normala Erromak beragatik ordaindutakoa. Italiako Erromarekin sinatu berri duen kontratuagatik harro dagoela esan zuen atzo [Gabriel Batistuta argentinarrak].
    “For [Batistuta] what Rome has paid for him is not normal. [The Argentinian Gabriel Batistuta] said yesterday that he is proud of the contract he recently signed recently with Rome, Italy.”
  (8) Bonba bat lehertu da [Londresen]. Eztandak ez du inor zauritu, eta kalte material txikiak eragin ditu, Hammersmith zubian, [Londres mendebaldean].
    “A bomb has exploded in [London]. The explosion did not hurt anyone and material damage have been small, in Hammersmith Bridge, in [west London].”
(S9) **Relaxed Head Match:** This sieve relaxes the entity head match constraint by allowing the mention head to match any word in the antecedent candidate cluster. Apart from this constraint, both mentions have to be named entities and the types must coincide. In addition, *word inclusion* and *i-within-i* constraints have to be fulfilled.
  **Problem:** The same problem that occurs with the *Entity Head Match* constraint arises when comparing the head words because word forms are used rather than lemmas. Moreover, as role appositives are not tagged in our corpus, they are not treated in the Precise Constructs sieve (S4). Nevertheless, they provide useful information that is needed to resolve some types of coreference relations that are treated in this sieve.
  **Adaptation**: To compare head words, lemmas are used instead of word forms. Furthermore, we have relaxed the constraint that needs both mentions to be named entities. In our case, only the antecedent mention has to be a proper noun, the candidate mention could be nominal. The last change has been adopted to link mentions with their antecedents that are in role appositive constructions.
  After the adaptation, the sieve considers the mentions [Portlandeko entrenatzaile Zupo Ekisoainekin] “with [Portland trainer Zupo Ekisoain]” and [Portlandeko entrenatzaileak] “[Portland trainer]” coreferent in example (9).
  (9) Gentzelek atzo goizean hitz egin zuen [Portlandeko entrenatzaile Zupo Ekisoainekin], Granollersek egindako eskaintza onartzeko arrazoiak azaltzeko. Granollersen lagunak ditu bere emazteak, eta hara joatea nahiago izan dutela azaldu zuen atzo [Portlandeko entrenatzaileak].
    “Gentzel talked with [Portland trainer Zupo Ekisoain] yesterday morning, to explain why he had accepted the offer made by Granollers. His wife has friends in Granollers, and they preferred to go there [the Portland trainer] explained yesterday.”
(S10) **Pronoun Resolution:** This sieve links pronominal mention with the antecedent mention based on these constraints:
  - Number: the number attribute is assigned based on:
    1. A static list of pronouns.
    2. A mention marked as a named entity is considered singular with the exception of organizations, which can be both singular and plural.
    3. NN*S part of speech tags are plural and other NN* tags are singular.
    4. A static dictionary.
  - Gender: gender attributes are assigned from static lexicons.
  - Person: person attributes are assigned to pronouns.
  - Animacy: Aanimacy is determined using: i) a static list of pronouns; ii) NER labels, e.g., PERSON is animate whereas LOCATION is not; iii) a dictionary bootstrapped from the web.
  - The NER label.
  - Pronoun distance: distance between a pronoun and its antecedent cannot be more than 3 sentences.
  **Problem:** Basque pronouns do not provide information about gender or animacy. The demonstrative determiners also act as third person pronouns.
  **Adaptation:** We have translated the static list of pronouns into Basque. We have enriched the static lexicon with 3,109 entries giving information about the gender of Basque, French and Spanish names. The pronoun distance constraint has been reduced to 2, i.e., the distance between a pronoun and its antecedent cannot be more than 2 sentences away. The modification of the pronoun distance value was optimised experimentally. As pronouns in Basque do not provide information about their gender and animacy they are more ambiguous than in other languages which are able to exploit those features. Consequently, the distance between the antecedent and the pronoun must be shorter. Taking this into consideration, we have changed the mention candidate selection algorithm so that the antecedent candidates for pronouns are sorted right-to-left traversal in the same and previous sentences in order to favour proximity. Finally, to improve the precision of the Pronoun resolution sieve, we apply a new constraint apart from just described. The definiteness of both mentions must match in order to consider them coreferent. After the modifications, the sieve is able to correctly link pronouns with their antecedents as in example (10).
  (10) Partidaren hastapenean Miarritzeko gazte euskaldun batzuek [euskal presoen aldeko zapiak] zeramatzaten bizkarrean, eta [haiekin] sartzen saiatu ziren.
    “At the beginning of the match some young Basques from Biarritz wore [scarves in favor of Basque prisoners] on their backs and tried to enter with [them].”
(S11) **Ellipsis Match:** In our corpus, elliptic mentions, i.e., structures in which ellipsis of a noun ellipsis occurs and suffixes attached to the word correspond to a noun, even when the noun is not explicit in the word, are tagged and can be part of coreference chains. The Stanford system does not have any sieve to treat elliptic mentions, so this sieve has been added to the coreference resolution system to treat cases of ellipsis.
  The sieve links a mention with an elided noun with its antecedent. To link two mentions the candidate mention and the candidate antecedent must agree in number and definiteness and they must appear in the same sentence. For example, the mention with elided noun [kalitate handikoak] in example (11) is linked with its antecedent [Biak].
  (11) Argentinako bi jokalari etorri ziren Gasteizera orain dela hamar urte: Nicola eta Guinazu. [Biak] oso gazteak ziren arren, [kalitate handikoak] ziren, eta etorkizun oparoa zuten.
    “Two Argentinian players came to Gasteiz ten years ago: Nicola and Guinazu. Although [both] were very young, they were [quality players], and they had a promising future.”

**Table 1:** Examples to illustrate the suitability of the Exact Morphology Match sieve.

#	Mention	Translation	Lemmas	Number	Definiteness	Coreferent
1	txori politak	pretty birds	txori polit	plural	definite	-
2	txori politekin	with the pretty birds	txori polit	plural	definite	yes
3	txori politak	pretty bird	txori polit	singular	definite	no
4	txori politek	pretty birds	txori polit	plural	indefinite	no

To sum up: in the version of the Stanford system adapted to Basque we make use of deeper morphological information to tackle the agglutinative nature of Basque. Changes related to free word order of Basque have also been implemented in the Basque system. We replaced one existing sieve with a new one, substituting the Exact String Match sieve with the Exact Morphology Match sieve, modified nine existing sieves and introduced one new sieve, Ellipsis Match.

## System evaluation

In this section, we evaluate the adapted Coreference resolution system. Several experiments have been carried out to measure different aspects of the system: i) for a baseline we applied a copy of the original Stanford Coreference resolution system for English using as input the output of the Basque linguistic processors and the Basque pronoun, demonym and gender lists; ii) our adapted system, EUSKOR, is compared with the baseline system. The two systems are compared using both automatic and gold mentions to identify the effect of preprocessing and mention detection on the results. iii) In order to obtain an optimal sieve order, both intuition-guided *hand-built ordering* and automatically obtained *learned ordering* are considered, iv) an incremental evaluation of the adapted system was performed to measure the contribution of individual sieves.

### Corpus

We divided the EPEC corpus into two parts: one for developing the system and another for testing. More detailed information about the two parts can be found in Table 2.

**Table 2:** EPEC corpus division information.

	Words	Mentions	Clusters	Singletons
Devel	30434	8432	1313	4383
Test	15949	4360	621	2445

### Metrics

The metrics used to evaluate the performance of the systems are MUC [49], *B*^3^ [50], CEAF_*e*_ [51], CEAF_*m*_ [51], and BLANC [52]. The CoNLL metric is the arithmetic mean of MUC, *B*^3^ and CEAF_*e*_ metrics. Scores have been calculated using the reference implementation of the CoNLL scorer [53]. The predictions used for calculating the scores can be downloaded at: http://ixa2.si.ehu.eus/asoraluze/EUSKOR/results/.

### Results

Table 3 shows the *F*_1_ scores obtained by the baseline (original Stanford system with Basque preprocessing and translated static lists) and EUSKOR system when automatically identified mentions were used. EUSKOR outperforms the baseline system according to *F*_1_ on all the metrics. In the CoNLL metric, EUSKOR has a score of 55.74, 7.07 points higher than the baseline system, which scores 48.67.

**Table 3:** Performance of baseline and EUSKOR systems with automatic mentions.

Automatic Mention Detection																
	MUC			*B*^3^			*CEAF*_*m*_			*CEAF*_*e*_			BLANC			CoNLL
	R	P	*F*_1_	R	P	*F*_1_	R	P	*F*_1_	R	P	*F*_1_	R	P	*F*_1_	*F*_1_
Baseline	22.48	35.27	27.46	54.81	66.17	59.96	56.13	57.6	56.86	62.08	55.50	58.61	33.47	44.96	36.75	48.67
EUSKOR	34.10	55.76	42.32	57.98	68.83	62.94	60.78	62.31	61.54	66.02	58.41	61.98	38.41	53.57	43.18	55.74

Scores obtained when gold mentions are provided are shown in Table 4. As we observe, the adapted system again outperforms the baseline according to all the metrics. The official CoNLL metric is outperformed by 11.5 points.

**Table 4:** Performance of baseline and EUSKOR systems with gold mentions.

Gold Mention Detection																
	MUC			*B*^3^			*CEAF*_*m*_			*CEAF*_*e*_			BLANC			CoNLL
	R	P	*F*_1_	R	P	*F*_1_	R	P	*F*_1_	R	P	*F*_1_	R	P	*F*_1_	*F*_1_
Baseline	31.6	43.32	36.55	76.32	86.92	81.28	72.13	72.13	72.13	80.44	72.11	76.05	59.47	71.06	62.94	64.62
EUSKOR	48.76	71.94	58.12	81.35	93.47	86.99	80.57	80.57	80.57	89.00	78.24	83.27	67.09	84.65	72.77	76.12

To isolate the behaviour of reference resolution from preprocessing and mention detection, it is interesting to observe the difference between the results obtained with automatic mentions (Table 3), gold mentions (Table 4) and gold boundaries (Table 5) are provided. It is clear that having accurate preprocessing tools and a good mention detector are crucial to obtaining good results in coreference resolution. The difference in CoNLL is about 15.95 points higher for the baseline system and 20.38 points higher for the adapted system when gold mentions are used. The scores behave similarly when perfect boundaries of the mentions are provided and the preprocessing is automatic, i.e., when gold boundaries of mentions are used.

**Table 5:** Performance of baseline and EUSKOR systems with gold mention boundaries.

Gold Mention Boundaries																
	MUC			*B*^3^			*CEAF*_*m*_			*CEAF*_*e*_			BLANC			CoNLL
	R	P	*F*_1_	R	P	*F*_1_	R	P	*F*_1_	R	P	*F*_1_	R	P	*F*_1_	*F*_1_
Baseline	31.39	43.97	36.63	76.39	87.39	81.52	72.53	72.53	72.53	81.16	72,36	76.51	59.45	71.48	62.99	64.88
EUSKOR	48.91	70.58	57.78	81.43	93.05	86.85	80.53	80.53	80.53	88.77	78.52	83.33	67.06	83.92	72.59	75.98

### Sieve ordering

The ordering of the sieves in the adapted system follows the same intuition as in the original Stanford Coreference Resolution system: firstly the most precise sieves are applied, then those that are less precise.

Nevertheless, since this order might not be optimal for Basque coreference resolution, we carried out an experiment to obtain the best sieve order automatically. A greedy search was used, and the best precision sieve at each stage was chosen. Tuning of the sieve order was achieved using the development part of the EPEC corpus and then evaluated using the test part.

Table 6 illustrates the new *learned ordering* proposed by the optimisation algorithm in comparison to the *hand-built ordering* represented in Fig 2.

**Table 6:** Hand-built ordering and Learned ordering.

Hand-built ordering	Learned ordering
S1 Speaker Identification	S1 Speaker Identification
S2 Exact Morphology Match	S11 Ellipsis Match
S3 Relaxed String Match	S2 Exact Morphology Match
S4 Precise Constructs	S3 Relaxed String Match
S5 Strict Head Match A	S4 Precise Constructs
S6 Strict Head Match B	S8 Proper Head Word Match
S7 Strict Head Match C	S6 Strict Head Match B
S8 Proper Head Word Match	S5 Strict Head Match A
S9 Relaxed Head Match	S7 Strict Head Match C
S10 Pronoun Resolution	S10 Pronoun Resolution
S11 Ellipsis Match	S9 Relaxed Head Match

The optimization resulted in some variations in all the metric scores although the CoNLL *F*_1_ remains at 55.74 points when automatic mentions are used.

### Incremental addition of sieves

In order to quantify the contribution of each individual sieve, we evaluated our system by adding 11 sieves incrementally. The sieves were added using the new Learned ordering proposed by the optimization algorithm. The results obtained are presented in Table 7.

**Table 7:** Performance of EUSKOR when sieves are added incrementally.

	MUC			*B*^3^			*CEAF*_*m*_			*CEAF*_*e*_			BLANC			CoNLL
	R	P	*F*_1_	R	P	*F*_1_	R	P	*F*_1_	R	P	*F*_1_	R	P	*F*_1_	*F*_1_
Speaker Identification	0	0	0	50.62	74.78	60.37	53.24	54.58	53.9	67.47	48.54	56.46	27	27.39	27.19	38.94
Ellipsis Match	0.15	22.22	0.3	50.65	74.7	60.37	53.29	54.63	53.95	67.47	48.64	56.53	27.03	38.5	27.25	39.06
Exact Morphology Match	23.56	73.96	35.74	55.44	73.29	63.13	59.32	60.82	60.06	68.29	54.43	60.58	34.65	64.76	39.95	53.15
Relaxed String Match	23.95	72.7	36.03	55.5	73.17	63.12	59.35	60.84	60.08	68.2	54.56	60.62	34.75	63.82	40.04	53.25
Precise Constructs	23.95	71.19	35.84	55.5	73.02	63.06	59.28	60.77	60.01	68.11	54.61	60.62	34.75	62.56	39.96	53.17
Proper Head Word Match	26.43	68.61	38.16	56.06	72.52	63.24	59.9	61.41	60.65	68.15	55.57	61.22	35.68	58.95	40.88	54.20
Strict Head Match B	28.91	65.66	40.15	56.64	71.89	63.36	60.41	61.93	61.16	67.87	56.42	61.61	36.43	58.42	41.74	55.04
Strict Head Match A	28.91	65.66	40.15	56.64	71.89	63.36	60.41	61.93	61.16	67.87	56.42	61.61	36.43	58.42	41.74	55.04
Strict Head Match C	30.07	63.39	40.79	56.92	71.34	63.32	60.39	61.91	61.14	67.31	56.63	61.51	36.87	57.84	42.18	55.20
Pronoun Resolution	32.24	58.42	41.55	57.52	69.68	63.02	60.53	62.05	61.28	66.56	57.6	61.76	37.77	54.66	42.73	55.44
Relaxed Head Match	34.1	55.76	42.32	57.98	68.84	62.95	60.76	62.28	61.51	66.00	58.4	61.97	38.41	53.35	43.14	55.74

The analysis of results reveals that the most significant improvements are due to the Exact Morphology Match sieve, which accounts for an improvement of 14.08 CoNLL *F*_1_ points, proving that the substitution of this for the original Exact String Match, and thus taking into account the morphological characteristics of the mentions, is necessary for morphologically rich languages.

The second greatest improvement in performance, around 1 point in CoNLL *F*_1_, is produced by the Proper Head Word Match sieve, and that is followed by the Strict Head Match B sieve yields a 0.83 improvement.

There is no gain in scores when the Strict Head A sieve is applied. The reason for this is that Strict Head Match B is applied before Strict Head Match A sieve. Since Strict Head Match B is a relaxation of Strict Head Match A, all the mentions that the A variation should link are already resolved when B is applied.

Notice that the CoNLL result falls slightly (by 0.08 points) when the Precise Constructs sieve is applied. This drop is caused by the predication and apposition structure identifier which does not always correctly identify these types of structures.

The results show that linking elliptical mentions with their antecedent is a complex task. Improving the CoNLL score for the Ellipsis Match sieve is low.

## Error analysis

Evaluation scores can show how efficient a coreference resolution system is; however, they neither identify the type of errors that it makes, nor give any indication of how those errors might be corrected.

A deep error-analysis can reveal the weak points of the coreference resolution system and help to decide future directions in the improvement of the system.

### Error causes

We present the classification of the error causes we found during the error analysis:

- **Preprocessing (PP):** Automatic tools used at preprocessing step (lemmatiser, PoS tagger, Named Entity recogniser etc.) make errors that provoke incorrect or missing links in coreference resolution.
- **Mention Detection (MD):** Errors made during mentions detection step, such as, missed mentions or incorrectly identified ones (incorrect identification of boundaries, not a mention…) are classified in this category.
- **Pronominal Resolution (PR):** A pronoun is linked with an incorrect antecedent.
- **Ellipsis Resolution (ER):** It is complicated to link elliptical mentions with their antecedent, as elliptical mentions omit the noun. Consequently, they do not provide much information to decide correctly which is their antecedent to be linked.
  For example, it is hard to link the elliptical mention [Yosi Beilin Israelgo Justizia ministroak Jeruralemi buruz esandako-Ø-ak] “what Yosi Beilin Israel Justice Minister said” (Ø refers to “what”) with its antecedent [Beilin Justizia ministroaren hitzak] “Beilin Justice minister’s words”.
- **Semantic Knowledge (SK):** Errors caused because the system is not able to resolve correctly the semantic relations (synonymy, hyperonymy, metonymy…) that happen between the heads of two coreferent mentions.
  For example, the different head words *parlamentua* and *legebiltzarra* of the mentions [Libanoko Parlamentuak] and [Libanoko Legebiltzarrak] (both mentions are translated as “Lebanon parliament”), are synonyms.
- **World Knowledge (WK):** In some cases world knowledge is required to resolve that two mentions are coreferent.
  For example, to link the mention [Reala] “Reala” with the mention [talde txuri-urdinak] “white-blue team”, it is necessary to know that *Reala* is a team and the nickname of the football team is *txuri-urdinak* “white-blue”. Humans normally are able to associate these types of mentions easily, but in the case of automatic systems world knowledge must be provided.
- **Miscellaneous (MISC):** Errors that are not contained in the above categories are classified in this category.
  For example, the mention [Kelme, Euskaltel eta Lampre] should be linked with the mention [Hiru taldeak] “The three teams”. In this specific example it is necessary to know that Kelme, Euskaltel and Lampre are teams and the enumerated mention has three elements.

After analysing the error causes done by our coreference resolution system in EPEC corpus we concluded that mention detection is crucial for coreference resolution, because 52.52% of errors caused by errors done in thin step. Improving mention detection would likely improve the scores obtained in coreference resolution. Nevertheless, in order to identify deficiencies of a coreference resolution system, Pronominal Resolution (9.17%), Ellipsis Resolution (3.21%), Semantics (6.42%) and World Knowledge (9.86%) categories can reveal how the errors might be corrected. Due to the variety of errors classified in miscellaneous category, little improvement would be achieved despite making a big effort to solve them.

Among all the error causes, in the next section we are going to focus on solving the errors provoked by the lack of semantic and world knowledge.

## Improving coreference resolution with semantic knowledge sources

This section explains the improvement process of EUSKOR integrating semantic knowledge sources presented more in detail in [54].

In order to treat cases where knowledge is needed, two new specialised sieves have been added to the coreference resolution system: One to extract knowledge from Wikipedia (Wiki-alias sieve) and the other to obtain semantic information from WordNet (Synonymy sieve).

(S12) **Wiki-alias sieve:** The new *Wiki-alias sieve* uses the mentions enriched by information obtained from Wikipedia pages.
  Named-entity mentions are linked with a Wikipedia page that UKB [55] says it is the most probable is used to enrich the mention.
  Using this information, the Wiki-alias sieve assumes that two mentions are coreferent if one of the three following conditions is fulfilled:
  1. the set of enriched word lemmas in the potential antecedent has all the mention candidate’s span lemmas.
  2. the head word lemma of the mention candidate is equal to the head word lemma of the potential antecedent or equal to any lemma in the set of enriched lemmas of the potential antecedent.
  3. all the enriched lemmas of the potential antecedent appear in the cluster lemmas of the mention candidate.
  This sieve considers coreferent the mentions *Osasunak* and *Taldeak* “{team}” and *gorritxoek* “{the reds}’ in (12), as the potential antecedent *Osasunak* has all the lemmas in the mention candidate’s span which after being enriched with {talde, futbol talde, gorritxo} “{team, football team, the reds}” linking to its Wikipedia page.
  (12) [Osasunak] lehenengo mailara igotzeko lehian azken astean bizi duen giroa oso polita da. [Taldea] lasaitzeko asmoz Oronozera eraman zituen Lotinak atzo guztiak. Oronozko kontzentrazioa beharrezkoa dute [gorritxoek].
    “[Osasuna] is going through a beautiful moment in the last week in the race to ascend to the Premier League. In order to reassure [the team] Lotina has decided to give all of them to Oronoz. [The reds] need to concentrate in Oronoz.”
(S13) **Synonymy sieve:** The *Synonymy sieve* uses the synonyms’ static list that we have extracted from Basque WordNet [56]. This sieve considers two mentions as coreferent if the following constraints are fulfilled:
  1. the head word of the potential antecedent and the head word of the mention candidate are synonyms
  2. all the lemmas in the mention candidate’s span are in the potential antecedent cluster word lemmas or *vice versa*.
  For example, in 13 the mention candidate *Libanoko legebiltzarra* and the *Libanoko parlametua* (both mentions are translated as “Lebanon parliament”) are considered coreferent as the different head words *legebiltzarra* and *parlamentua* are synonyms and the lemma *Libano* “Lebanon” of the word *Libanoko* is present in the cluster word lemmas of the potential antecedent.
  (13) [Libanoko Parlamentuak] batzar historikoa gauzatu du askapenaren martiriak omentzeko. Askapenaren martiriak goratu zituen atzo [Libanoko Legebiltzarrak], batzar historiko baten bitartez.
    “[The Lebanese Parliament] has made a historic assembly to pay tribute to the martyrs of liberation. [The Lebanese Parliament] exalt the martyrs of liberation yesterday, through a historic assembly.”

In order to quantify the impact of using semantic knowledge sources in coreference resolution, we have tested the enriched coreference resolution system using the EPEC corpus and compared the results with those obtained by EUSKOR. The experimentation has been carried out using automatic mentions (*F*_1_ = 77.57) and gold mentions (*F*_1_ = 100). The results are presented in Table 8 and in Table 9 respectively.

**Table 8:** Results obtained when automatic mentions are used. 1=EUSKOR, 2=1+Wiki sieve, 3=1+Synonymy sieve, 4=1+Wiki sieve+Synonymy sieve.

Automatic mentions																
	MUC			*B*^3^			*CEAF*_*m*_			*CEAF*_*e*_			BLANC			CoNLL
	R	P	*F*_1_	R	P	*F*_1_	R	P	*F*_1_	R	P	*F*_1_	R	P	*F*_1_	*F*_1_
1	34.1	55.76	42.32	57.98	68.83	62.94	60.78	62.31	61.54	66.02	58.41	61.98	38.41	53.57	43.18	55.74
2	34.41	55.70	42.54	58.09	68.64	62.93	60.73	62.26	61.49	65.94	58.49	61.99	38.65	53.27	43.35	55.82
3	34.57	56.03	42.76	58.08	68.80	62.98	60.85	62.38	61.61	65.99	58.51	62.03	38.53	53.65	43.31	55.92
4	34.88	55.90	42.95*	58.19	68.60	**62.97**	60.80	62.33	**61.56**	65.92	58.60	**62.04**	38.77	53.33	43.48*	55.98*

**Table 9:** Results obtained when gold mentions are used. 1=EUSKOR, 2=1+Wiki sieve, 3=1+Synonymy sieve, 4=1+Wiki sieve+Synonymy sieve.

Gold mentions																
	MUC			*B*^3^			*CEAF*_*m*_			*CEAF*_*e*_			BLANC			CoNLL
	R	P	*F*_1_	R	P	*F*_1_	R	P	*F*_1_	R	P	*F*_1_	R	P	*F*_1_	*F*_1_
1	48.76	71.94	58.12	81.35	93.47	86.99	80.57	80.57	80.57	89.00	78.24	83.27	67.09	84.65	72.77	76.12
2	49.84	70.81	58.50	81.71	92.83	86.92	80.57	80.57	80.57	88.69	78.77	83.44	67.51	83.27	72.84	76.28
3	50.00	71.50	58.85	81.69	93.19	87.06	80.80	80.80	80.80	88.90	78.82	83.56	67.39	84.23	72.95	76.49
4	50.46	70.99	58.99*	81.86	92.81	86.99	80.71	80.71	**80.71**	88.71	79.00	83.57*	67.68	83.34	**73.00**	76.51*

Observing the results presented in Table 8, we can see that the EUSKOR’s *F*_1_ scores are outperformed in all the metrics by the semantically enriched system. In CoNLL metric, the improved system has a score of 55.98, which is slightly higher than EUSKOR, to be precise, 0.24 higher.

As shown in Table 9, EUSKOR *F*_1_ scores are also outperformed in all the metrics, except in *B*^3^ when gold mentions are used. The official CoNLL metric is improved by 0.39 points (*F*_1_ = 76.51).

It is interesting to compare the improvements in CoNLL score obtained by the system after adding two new sieves. The improvement when automatic mentions are used is lower than when gold mentions are provided, 0.24 and 0.39 respectively. In both cases, even the improvements obtained are modest, they are statistically significant using Paired Student’s t-test with p-value < 0.05. Statistically significant values are marked with * symbol.

As pointed out in [21], despite the fact that absolute performance numbers are much higher on gold mentions and there is less room for improvement, the semantic features help much more than they do in system mentions. Similar idea is added in [57], as they mention that in realistic settings, where the loss in precision would be amplified by the additional non-gold mentions, it is substantially harder to achieve gains by incorporate lexical and encyclopedic knowledge, but possible and necessary.

## Comparison of coreference systems for Basque

Finally, in this section EUSKOR is compared with a learning-based system and a neural system for Basque coreference resolution. The results obtained by the three systems are directly comparable as the corpus used for evaluation is the same, the EPEC corpus.

The learning-based system is BART [28], a modular toolkit for coreference resolution that was extended in order to enable it to process Basque [58]. More concretely, two different models have been trained, a baseline model based on [59] and a Basque model with extended feature set. An additional independent Language Plugin module for Basque than handles language specific information has been also developed.

Apart from the learning-based system, recently, a first neural cross-lingual coreference resolution system for Basque has been presented [60]. This system learns from a English corpus consisted of around 825K words and 100K mentions (much bigger than EPEC) using cross-lingual embeddings to perform coreference resolution for Basque.

In order to compare different approaches (neural vs. learning-based vs. rule-based) to Basque coreference resolution, we looked at the results obtained by the three systems that are presented in Table 10. The results obtained with learning-based and neural systems are lower than those obtained by EUSKOR, the rule-based system presented in this paper. EUSKOR is seen to have better F-scores values than BART’s and neural cross-lingual system in all the metrics, with 2.02 point and 14.81 advantage in the CoNLL metric.

**Table 10:** Comparison of performance of adapted Stanford and BART systems.

Automatic Mention Detection						
	MUC	*B*^3^	*CEAF*_*m*_	*CEAF*_*e*_	BLANC	CoNLL
	F1	F1	F1	F1	F1	F1
EUSKOR	**42.32**	**62.94**	**61.54**	**61.98**	**43.18**	**55.74**
BART	39.86	61.48	59.38	59.84	42.41	53.72
NEURAL	8.30	58.61	53.37	55.87	29.14	40.93

Taking into consideration that EUSKOR beats the other systems, we conclude that EPEC corpus, consisted of 45,000 words, is too small for learning-based and neural systems to learn properly as they do with big corpora. Surely, increasing the size of the corpus would enhance the results obtained by learning-based and neural-based systems.

## Conclusions and future work

We adapted the Stanford Coreference Resolution system to the Basque language and integrated it into a global architecture of linguistic processors to obtain EUSKOR, an end-to-end coreference resolution system. Taking into account the characteristics of Basque, we have described the adaptation process in detail, sieve by sieve, to facilitate the replicability of the process for other languages. The adapted system was evaluated comparing the results with a baseline system (original Stanford system with Basque preprocessing and translated static lists) in two scenarios, automatic mentions and gold mentions. The adapted system outperforms the baseline in all metrics. In CoNLL *F*_1_ when automatic mentions are used the baseline is surpassed by 7.07 points and by 11.5 points when gold mentions are used. We also carried out an incremental experiment to quantify the contribution of each individual sieve and we have performed a deep error analysis which have revealed our system’s weak points and help to determine future directions in the improvement of the system. As a result of the error analysis, we have enriched the Basque coreference resolution adding new two sieves, *Wiki-alias* and *Synonymy sieve*, obtaining an improvement of 0.24 points when automatic mentions are used and of 0.39 points using gold mentions.

The results obtained by our system were compared with a learning-based and a neural system Basque coreference resolution, and it was shown that ruled-based system obtains better results in all the metrics. In CoNLL metric, EUSKOR obtains an advantage of 2.22 points compared to BART’s score, and the difference of 14.81 points compared to neural-based system. We have concluded that EPEC corpus’ size is too small for learning-based and neural systems to learn properly.

As future work, we intend to improve pronoun resolution and ellipsis resolution, as we observed in the error analysis presented that they are the cause of considerable coreference resolution errors, around 12% of total errors. It would also be interesting to investigate other types of techniques such as machine learning (if the EPEC corpus is increased) and combine the strengths of rule-based methods and learning-based methods in a hybrid approach similar to [15]. In addition, we would like to further investigate neural approaches for coreference resolution.
